# AβPP processing results in greater toxicity per amount of Aβ_1-42_ than individually expressed and secreted Aβ_1-42_ in *Drosophila melanogaster*

**DOI:** 10.1242/bio.017194

**Published:** 2016-07-07

**Authors:** Liza Bergkvist, Linnea Sandin, Katarina Kågedal, Ann-Christin Brorsson

**Affiliations:** 1 Division of Molecular Biotechnology, Department of Physics, Chemistry and Biology, Linköping University, Linköping 58183, Sweden; 2 Division of Cell Biology, Department of Clinical and Experimental Medicine, Faculty of Medicine and Health Sciences, Linköping University, Linköping 58183, Sweden

**Keywords:** Alzheimer's disease, Amyloid-beta (Aβ), AβPP processing, *Drosophila melanogaster*, Proteotoxicity

## Abstract

The aggregation of the amyloid-β (Aβ) peptide into fibrillar deposits has long been considered the key neuropathological hallmark of Alzheimer's disease (AD). Aβ peptides are generated from proteolytic processing of the transmembrane Aβ precursor protein (AβPP) via sequential proteolysis through the β-secretase activity of β-site AβPP-cleaving enzyme (BACE1) and by the intramembranous enzyme γ-secretase. For over a decade, *Drosophila melanogaster* has been used as a model organism to study AD, and two different approaches have been developed to investigate the toxicity caused by AD-associated gene products *in vivo*. In one model, the Aβ peptide is directly over-expressed fused to a signal peptide, allowing secretion of the peptide into the extracellular space. In the other model, human AβPP is co-expressed with human BACE1, resulting in production of the Aβ peptide through the processing of AβPP by BACE1 and by endogenous fly γ-secretase. Here, we performed a parallel study of flies that expressed the Aβ_1-42_ peptide alone or that co-expressed AβPP and BACE1. Toxic effects (assessed by eye phenotype, longevity and locomotor assays) and levels of the Aβ_1-42_, Aβ_1-40_ and Aβ_1-38_ peptides were examined. Our data reveal that the toxic effect per amount of detected Aβ_1-42_ peptide was higher in the flies co-expressing AβPP and BACE1 than in the Aβ_1-42_-expressing flies, and that the co-existence of Aβ_1-42_ and Aβ_1-40_ in the flies co-expressing AβPP and BACE1 could be of significant importance to the neurotoxic effect detected in these flies. Thus, the toxicity detected in these two fly models seems to have different modes of action and is highly dependent on how and where the peptide is generated rather than on the actual level of the Aβ_1-42_ peptide in the flies. This is important knowledge that needs to be taken into consideration when using *Drosophila* models to investigate disease mechanisms or therapeutic strategies in AD research.

## Abstract (precis)

**Summary:** In *Drosophila*, the proteotoxic effect of Aβ_1-42_ is highly dependent on how and where the peptide is generated, rather than on the peptide level in the flies, with implications for Alzheimer's disease research.

## INTRODUCTION

Alzheimer's disease (AD) is a neurodegenerative disease that gradually destroys brain cells and leads to progressive decline in mental function. It is the most prevalent form of dementia, affecting 11% of the population over the age of 65, and the sixth leading cause of death in the US (Thies and Bleiler, 2013). As human longevity increases, AD will affect a larger number of people and will involve huge economic costs associated with the need to care for suffering individuals. Thus, the requirement to find an effective treatment for the disease is urgent. Aggregation of amyloid-β (Aβ) peptides into fibrillar deposits known as amyloid plaques has long been considered the key neuropathological hallmark of AD (Hardy and Higgins, 1992). Aβ peptides are generated by proteolytic processing of the transmembrane Aβ precursor protein (AβPP) through sequential proteolysis by the β-secretase activity of β-site AβPP-cleaving enzyme (BACE1) and by the intramembranous enzyme complex γ-secretase (De Strooper and Annaert, 2000). Depending on the site of cleavage, different-sized Aβ peptides are generated, where Aβ_1-40_ and Aβ_1-42_ are the most frequent Aβ isoforms. Aβ_1-42_ has a higher propensity to form prefibrillar aggregates compared to Aβ_1-40_ and has also been reported to be more toxic than Aβ_1-40_ (Dahlgren et al., 2002).

To increase understanding of the different pathways and mechanisms involved in AD, appropriate disease models are necessary. Murine AD models, which are often based on overexpression of human AβPP, generate extracellular amyloid plaques characteristic of the disease (Games et al., 1995). However, many of the murine models do not display cognitive deficits or neurodegeneration, two important features of the disease pathology observed in humans (Bryan et al., 2009). Another model organism, which emerged in 2004 as a potential candidate for AD modelling, is *Drosophila melanogaster*. Two different *Drosophila* models of AD involving Aβ proteotoxicity have been generated and characterized. In one model, the gene encoding the Aβ peptide was cloned into the fly genome; the peptide was expressed fused to a signal sequence, allowing secretion of the peptide (Crowther et al., 2005; Finelli et al., 2004; Iijima et al., 2004). In the other model, human AβPP was co-expressed with human BACE1, allowing the production of different isoforms of the Aβ peptide (including post-translationally modified Aβ variants) through the processing of human AβPP by human BACE1 and by endogenous fly γ-secretase (Greeve et al., 2004). Several studies using one of these two approaches have been conducted to investigate toxic effects caused by these AD-associated gene products and to explore various therapeutic strategies, including feeding flies with substances that modulate the Aβ aggregation pathway or the processing of AβPP, the co-expression of Aβ with anti Aβ-toxicity proteins and genetic manipulation of cellular pathways involved in AD (Berg et al., 2010; Chakraborty et al., 2011; Crowther et al., 2005; Favrin et al., 2013; Helmfors et al., 2015; Hermansson et al., 2014; Luheshi et al., 2010; Rival et al., 2009).

To further understand the usefulness of these two *Drosophila* models to study the mechanisms of AD and to unveil the proteotoxic effects of the Aβ peptide, we investigated how the toxic effects might be linked to the level of Aβ_1-42_ and/or how the peptide is produced in the fly. To achieve this, we conducted a study where the toxic effects and levels of Aβ_1-42_ were examined in parallel in Aβ_1-42_-expressing flies and in flies that co-expressed human AβPP with human BACE1 (AβPP-BACE1 flies). In both fly models, the Gal4/UAS system was used to direct expression of the transgenes to post-mitotic neurons, using the *elav*-Gal4 driver (Yao and White, 1994), or to the retina, using the *gmr*-Gal4 driver (Moses et al., 1989). To probe toxicity, eye morphology was examined at the day of eclosion in *gmr*-Gal4-derived flies, and longevity and locomotor activity were assayed in *elav*-Gal4-derived flies. The levels of Aβ_1-42_ and of the BACE1-cleaved N-terminal AβPP product (AβPP_β_) were determined by Meso Scale Discovery (MSD) immunoassay, in the head of the *gmr*-Gal4-derived flies and separately in the head and body of the *elav*-Gal4-derived flies. To get a better picture of the *in vivo* AβPP processing in the AβPP-BACE1 flies, the levels of Aβ_1-40_ and Aβ_1-38_ were also analysed in the *elav*-Gal4-derived flies. Formation of amyloid aggregates in the brain was examined in both AD fly models. This study showed that the toxic effect per amount of detected Aβ_1-42_ in the fly was considerably higher in the AβPP-BACE1 flies compared to the Aβ_1-42_ flies, which reveals that Aβ_1-42_ proteotoxicity is highly dependent on how and where the peptide is generated rather than on the actual level of Aβ_1-42_ peptide in the flies. In addition, the co-existence of Aβ_1-42_ and Aβ_1-40_ detected in the AβPP-BACE1 flies could be of significant importance to the neurotoxic effect detected in these flies.

## RESULTS

### Generation of Aβ_1-42_ and AβPP-BACE1 flies

**Fig. 1.**
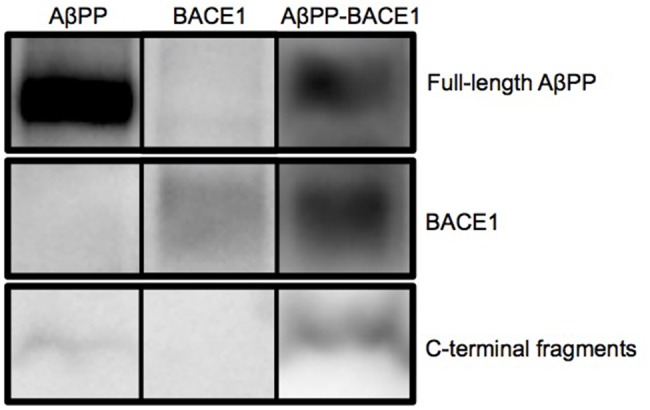
**Western blot analysis shows correct expression of transgenes.** Protein expression analysis of human AβPP and human BACE1 from *elav*-Gal4-derived flies. Lane 1: AβPP, lane 2: BACE1 and lane 3: AβPP- BACE1. *n*=4 (with 40 flies in each set). The areas of blots corresponding to the molecular weights of AβPP, BACE1 and the C-terminal fragments from the indicated flies are shown.

A fly line containing double copies of signal peptide Aβ_1-42_ (Aβ_1-42_×2 flies) was generated as previously described (Crowther et al., 2005). Transgenic fly lines containing the gene encoding human AβPP_695_ (AβPP) or human BACE1 were purchased from the Bloomington Stock Centre. The expression of AβPP and BACE1 under control of the *elav* promotor was examined by western blot analyses. Two transgenic fly lines that showed good production of AβPP and BACE1 were used to create a double transgenic AβPP-BACE1 fly line. From western blot analyses, full-length AβPP (∼100 kDa) was detected in the brains of flies that expressed AβPP alone (lane 1) or that co-expressed AβPP and BACE1 (lane 3) (Fig. 1). No AβPP was detected in the BACE1-expressing flies (lane 2). BACE1 (∼56 kDa) was detected in the BACE1-expressing flies (lane 2) and in the AβPP-BACE1-expressing flies (lane 3) (Fig. 1). Notably, for the AβPP-BACE1-expressing flies, a lower molecular band was detected (10-13 kDa). This band corresponds to AβPP C-terminal fragments (CTFs), which are products of full-length AβPP after cleavage by BACE1. Thus, processing of AβPP by BACE1 in the AβPP-BACE1-expressing flies was confirmed with this analysis.

### Co-expression of AβPP and BACE1 in the fly retina disturbs eye development

**Fig. 2.**
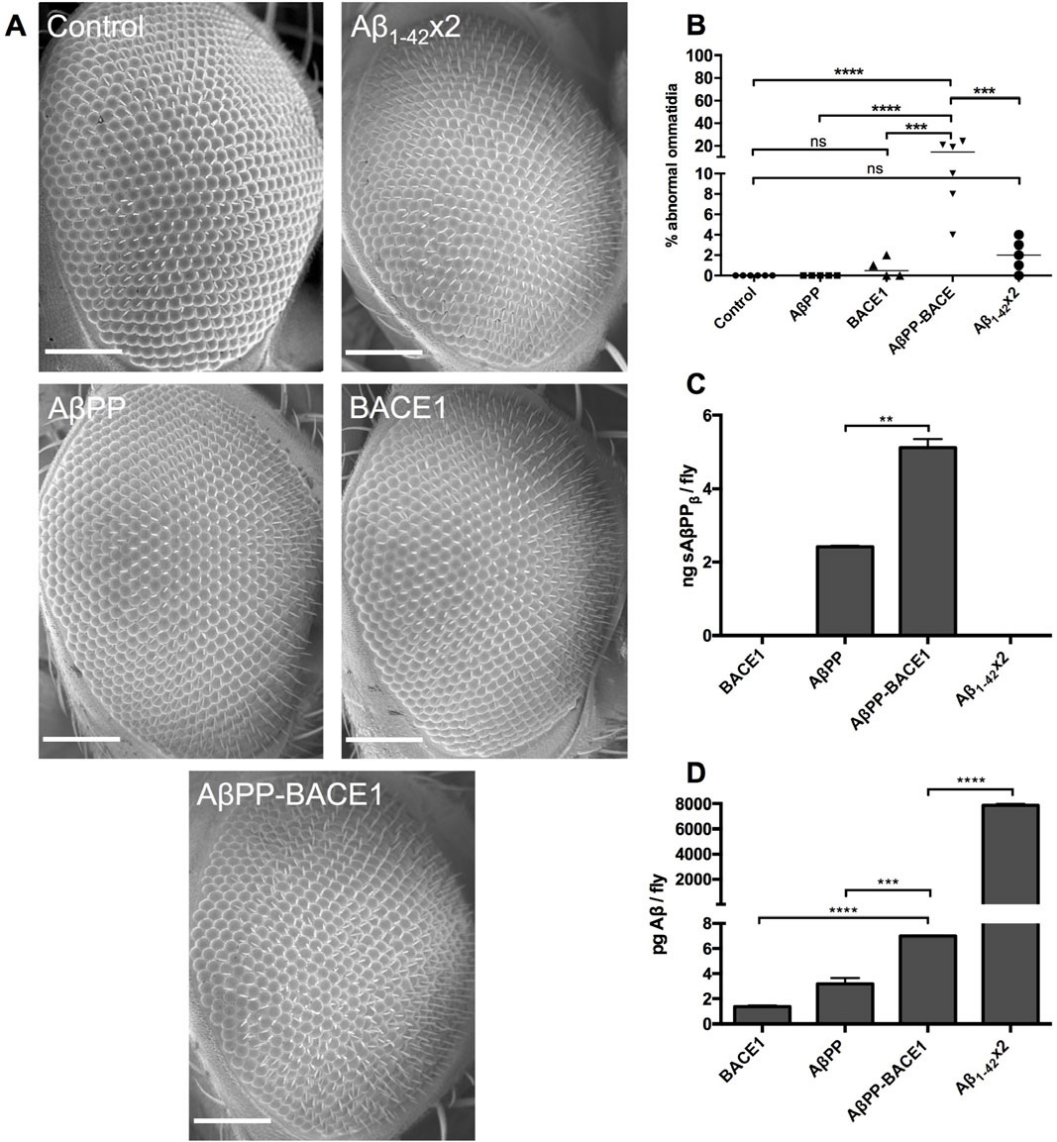
**The AβPP-BACE1 flies have a more pronounced rough eye phenotype and a lower level of Aβ_1-42_ compared to the Aβ_1-42_ flies.** (A,B) Toxic effects, by rough eye phenotype analysis (A), were assessed for *gmr*-Gal4-derived control, Aβ_1-42_×2, BACE1, AβPP and AβPP-BACE1 flies followed by (B) quantification of percentage of abnormal ommatidia, *n*≥4. Scale bar: 50 μm. (C,D) MSD analyses were performed for *gmr*-Gal4-derived flies to measure the levels of (C) sAβPP_β_ and (D) Aβ_1-42_ peptide. *n*=3 (with 20 flies in each set), **P≤0.01, ***P≤0.001, ****P≤0.0001. Data represented as mean±s.d.

Flies that expressed AβPP, BACE1, and Aβ_1-42_×2 individually or that co-expressed AβPP and BACE1 (AβPP-BACE1 flies) and control *w^1118^* flies (which only expressed Gal4) were analysed for toxic effects during eye development using the *gmr*-Gal4 driver, which drives the expression of transgenes in the fly retina (Moses et al., 1989). Flies were reared at 25°C, and the eyes of the offspring were examined using scanning electron microscopy at the day of eclosion (Fig. 2A). The disruption in the ommatidia was quantified by a blinded set-up. This was done by assigning the images of the eyes of each genotype with a square consisting of approximately 100 clearly visible and focused ommatidia in the centre of the eye. All ommatidia within this square were calculated and the number of abnormal ommatidia was related to the total number of ommatidia in the square. For the Aβ_1-42_-expressing flies a disruption in the eye structure could be detected for isolated ommatidia; however, the statistical analysis did not reveal any significant difference in the eye structure between the Aβ_1-42_ flies and control flies (Fig. 2B). No significant disruptions in the arrangement of ommatidia were detected for flies individually expressing AβPP or BACE1 compared to control flies (Fig. 2B). When analysing the eye structure of the AβPP-BACE1 flies, a significant disruption of the ommatidia arrangement was detected for these flies compared to the AβPP and BACE1 flies (*P*≤0.0001 and *P*≤0.001; Fig. 2B) and the Aβ_1-42_×2 flies (*P*≤0.001; Fig. 2B). These data indicate a more damaging effect on eye development in the AβPP-BACE1 flies compared to the Aβ_1-42_×2 flies.

### The level of Aβ_1-42_ in the AβPP-BACE1 flies is lower than that in the Aβ_1-42_ flies

Next, the presence of soluble N-terminal AβPP product (sAβPP_β_) and the Aβ_1-42_ peptide in the heads of the *gmr*-Gal4-derived fly variants were examined using the MSD technique. In the AβPP-BACE1 co-expressing flies, a sAβPP_β_ signal was detected that corresponded to a level of 5 ng/fly (Fig. 2C). This signal was significantly lower (*P*≤0.05) in the AβPP flies, in which it corresponded to a level of 2.4 ng/fly. No sAβPP_β_ signal was detected in the BACE1 or Aβ_1-42_×2 flies. In the Aβ_1-42_ analysis, high signal from the peptide was detected in the Aβ_1-42_×2 flies; the signal corresponded to 7900 pg/fly (Fig. 2D). The presence of Aβ_1-42_ was also detected in the AβPP-BACE1 flies at a level of 7 pg/fly. This level was significantly higher than the Aβ_1-42_ levels detected in the flies that expressed AβPP (*P*≤0.001) or BACE1 (*P*≤0.0001) individually (3.2 pg/fly and 1.4 pg/fly, respectively). These data confirm that AβPP was being correctly processed by BACE1 and fly γ-secretase in the AβPP-BACE1 flies to generate Aβ peptides, although the Aβ_1-42_ level detected in these flies was found to be significantly lower (*P*≤0.0001) than the level detected in the Aβ_1-42_×2 flies. In summary; these data show that although the AβPP-BACE1 flies had a lower level of Aβ_1-42_ compared to the Aβ_1-42_×2 flies, the AβPP-BACE1 flies displayed a higher toxic effect in the fly retina.

### The Aβ_1-42_ and AβPP-BACE1 flies exhibit toxic effects on fly neurons

**Fig. 3.**
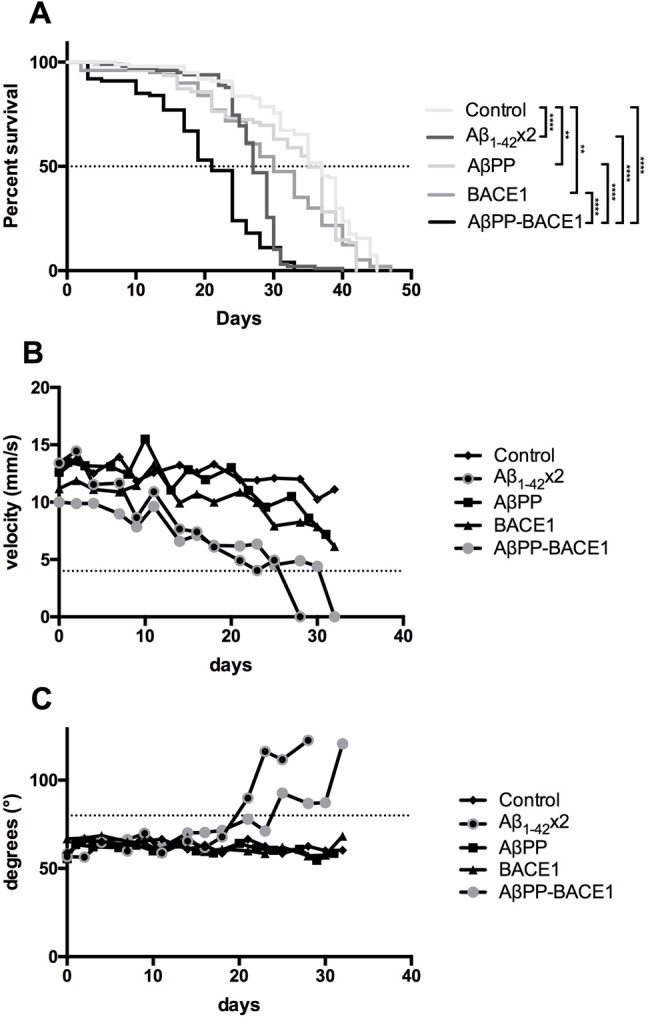
**Longevity and locomotor analyses show toxic effects on the neurons of the Aβ_1-42_ and AβPP-BACE1 flies.** Toxic effects were assessed for *elav*-Gal4-derived control, Aβ_1-42_×2, AβPP, BACE1 and AβPP-BACE1 flies by (A) longevity assay, (B) velocity measurement and (C) angle of movement analysis. *n*=100 for the longevity assay, and *n*=30 for the locomotor analyses, **P≤0.01 and ****P≤0.0001. Statistical analysis of survival data was performed on the median survival times.

Next, the transgenes were expressed in the central nervous system (CNS) of the fly using the *elav*-Gal4 driver, and the flies were analysed for longevity to monitor the toxic effects on fly neurons (Crowther et al., 2005; Hirth, 2010). Expressing the Aβ_1-42_ peptide in the fly neurons resulted in a 10-day (*P*≤0.0001) reduction in the median survival time (the day when 50% of the flies were dead) of the Aβ_1-42_×2 flies compared to the control flies; the median survival times were 27 days for the Aβ_1-42_×2 flies and 37 days for the control flies (Fig. 3A). For the AβPP-BACE1 flies, the median survival time was 21 days, resulting in a reduction of 14 days (*P*≤0.0001) and 9 days (*P*≤0.0001) compared to that for the AβPP flies and the BACE1 flies, respectively; the median survival times were 35 days for the AβPP flies and 30 days for the BACE1 flies (Fig. 3A). When the two different AD fly models were compared, the AβPP-BACE1 flies had a significant reduction in the median survival time (6 days) compared to the Aβ_1-42_×2 flies (*P*≤0.0001; Fig. 3A). Both AβPP flies and the BACE1 flies displayed a reduction in the median survival time (2 and 5 days, respectively) compared to control flies (*P*≤0.01, Fig. 3A).

To achieve a more complete picture of fly health, a locomotor assay was performed using the iFly technique (Jahn et al., 2011; Helmfors et al., 2015) to analyse the velocity and angle of movement. As flies age, their velocity decreases. Shortly before the flies die, they become immobile and their velocity cannot be recorded; thus, a cut-off value of 4 mm/s was set as an indication of disability. Both the AβPP-BACE1 and the Aβ_1-42_×2 flies showed a substantial reduction in velocity, falling below the cut-off value of 4 mm/s at day 32 and 28, respectively (Fig. 3B). This disability was not detected in the AβPP or BACE1 flies. Additionally, the angle of movement increases as the flies age. A cut-off value of 80° was set; values above this cut-off indicate that the mobility of the fly is impaired. In the angle of movement analysis, the AβPP-BACE1 and Aβ_1-42_×2 flies diverted from healthy control flies at days 26 and 22, respectively, when the cut-off value of 80° was reached. In contrast, the AβPP and BACE1 flies followed the control curve (Fig. 3C). Taken together, the longevity and locomotor analyses showed that co-expression of AβPP and BACE1 in the fly CNS causes toxic effects with similar magnitudes to those detected when expressing Aβ_1-42_ directly in the fly CNS.

### The toxic effect per amount of Aβ_1-42_ in the AβPP-BACE1 flies is higher than that in the Aβ_1-42_ flies

**Fig. 4.**
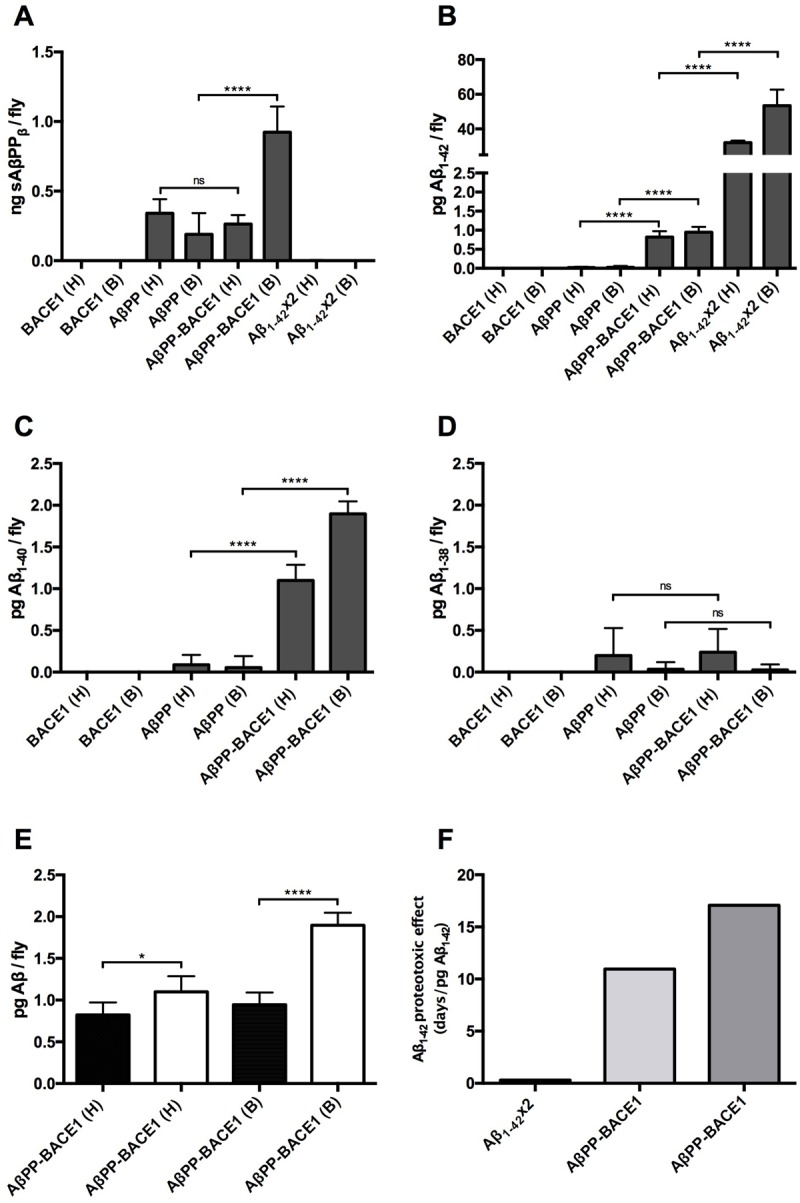
**The proteotoxic effect of Aβ_1-42_ is substantially stronger in the AβPP-BACE1 flies compared to the Aβ_1-42_ flies.** (A-D) MSD analyses were performed separately for the head and body of *elav*-Gal4-derived flies to measure the levels of (A) sAβPP_β_, (B) Aβ_1-42_ peptide (C) Aβ_1-40_ peptide and (D) Aβ_1-38_ peptide. (E) The levels of the Aβ_1-40_ peptide (white) were found to be significantly higher than the levels of the Aβ_1-42_ peptide (black) in both the head and the body of the AβPP-BACE1 flies. *n*=3 (with 20 flies in each set), ****P≤0.0001, *P≤0.05. (F) The Aβ_1-42_ proteotoxic effect was assessed by dividing the reduction in medium survival time of the Aβ_1-42_ flies relative to the control flies (black) and of the AβPP-BACE1 flies relative to the AβPP flies (grey) or BACE1 flies (dark grey), with the level of Aβ_1-42_ per fly head for the *elav*-Gal4-derived Aβ_1-42_ and AβPP-BACE1 flies. Data represented as mean±s.d.

The levels of sAβPP_β_ and Aβ_1-42_ were examined in the head and in the body of the *elav*-Gal4-derived fly variants using the MSD technique. The highest level of sAβPP_β_ was found in the body of the AβPP-BACE1 flies (0.92 ng/fly); this level was significantly higher (*P*≤0.0001) than the level detected in the body of the AβPP flies (0.18 ng/fly) (Fig. 4A). No sAβPP_β_ was detected in the body of the BACE1 or Aβ_1-42_ flies. In the sAβPP_β_ analyses of the fly heads, a weak sAβPP_β_ signal was found in both the AβPP and AβPP-BACE1 flies. In these two variants, the signal was similar in amplitude, whereas no signal was detected in the BACE1 or Aβ_1-42_ flies (Fig. 4A). In the total Aβ_1-42_ analysis (Fig. 4B), the highest levels of the peptide were found in the head and body of the Aβ_1-42_ flies; the head and body contained 32 pg/fly and 53 pg/fly, respectively. The levels of Aβ_1-42_ in the AβPP-BACE1 flies were found to be 0.82 pg/fly head and 0.95 pg/fly body. The Aβ_1-42_ signals detected in the head and body of the AβPP flies were weak, and no signals were detected in the head or body of the BACE1 flies.

To further investigate the *in vivo* processing of AβPP in the AβPP-BACE1 flies, the total levels of Aβ_1-40_ and Aβ_1-38_ were analysed (Fig. 4C,D). The detected levels of Aβ_1-40_ in the head and body of the AβPP-BACE1 flies were 1.1 pg/fly and 1.95 pg/fly, respectively. These levels were significantly higher than the Aβ_1-40_ signals found in the body and the head of the AβPP flies (*P*≤0.0001 and *P*≤0.001; Fig. 4C). No Aβ_1-40_ signal was detected for the BACE1 flies. When analysing the total levels of Aβ_1-38_, a weak signal was observed in the head and the body of both AβPP-BACE1 and AβPP flies. However, no significant difference was observed between the signals in AβPP-BACE1 flies compared to the signals in the AβPP flies. For the head and body of the BACE1 flies, no Aβ_1-38_ signals were detected. When the levels of Aβ_1-42_ and Aβ_1-40_ produced in the AβPP-BACE1 flies were compared, significantly higher levels of Aβ_1-40_ were detected in both the head (*P*≤0.05) and the body (*P*≤0.0001) compared to Aβ_1-42_ (Fig. 4E).

Taken together, these data revealed that, in line with the MSD analyses of the *gmr*-Gal4-derived fly variants, the level of Aβ_1-42_ was significantly lower in the AβPP-BACE1 flies compared to the Aβ_1-42_×2 flies; this was found both in the head (*P*≤0.0001) and in the body (*P*≤0.0001) of the flies. However, the toxic effect on the fly neurons of the AβPP-BACE1 and the Aβ_1-42_ flies, as assessed by the survival assay, was of the same magnitude (9 and 14 days reduction in the median survival for the AβPP-BACE1 flies compared to the AβPP and BACE1 flies, respectively, and 10 days reduction in the median survival for the Aβ_1-42_ flies compared to the control flies). This means that the toxic effect per amount of detected Aβ_1-42_ in the fly was considerably higher in the AβPP-BACE1 flies compared to the Aβ_1-42_ flies. This difference is visualized in Fig. 4F, where the Aβ_1-42_ proteotoxic effect (quantified as the reduction in the median survival divided by the amount of Aβ_1-42_ per fly head) is presented for the Aβ_1-42_ and the AβPP-BACE1 flies. Additionally, the MSD data also showed that a large portion of the Aβ_1-42_ detected in the AβPP-BACE1 and Aβ_1-42_×2 flies (54% and 62%, respectively) was transported from the brain to the body of the fly.

### The amount of amyloid aggregates is lower in the AβPP-BACE1 flies compared to the Aβ1-42 flies

**Fig. 5.**
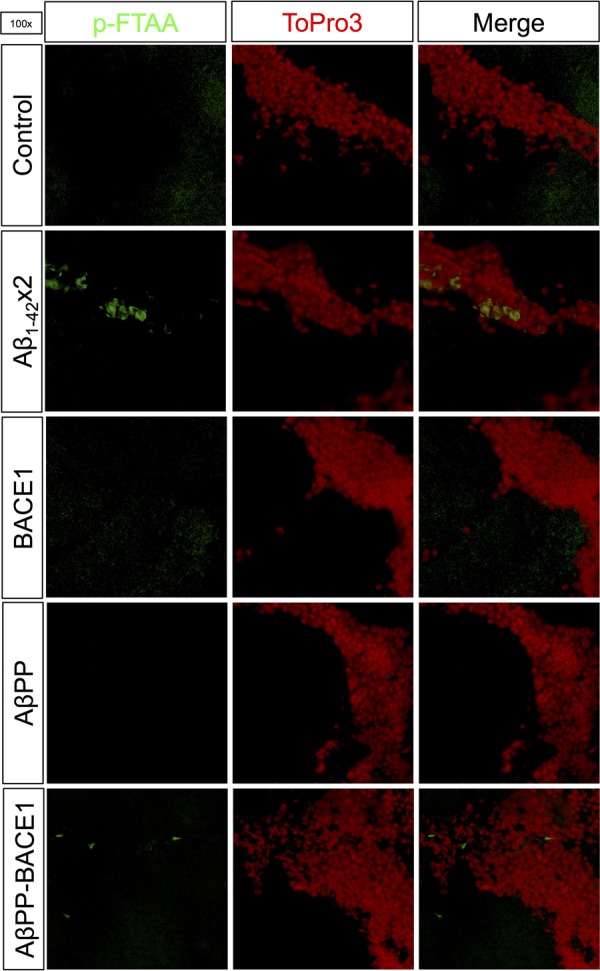
**A lower load of amyloid aggregates was detected in the AβPP-BACE1 flies compared to the Aβ_1-42_ flies.** *Drosophila* brain sections were stained with the amyloid-specific dye p-FTAA (green) and the nucleus stain ToPro3 (red). An extensive amount of amyloid aggregates were detected in Aβ_1-42_×2 flies, and a smaller amount was detected in the AβPP-BACE1 flies. No p-FTAA-positive signal could be detected in control, AβPP or BACE1 flies. Micrographs were taken at 100×, a minimum of eight fly brains were analysed for each genotype (representative micrographs are shown).

To investigate whether AD characteristic amyloid aggregates were formed in the Aβ_1-42_×2 and AβPP-BACE1 flies, *Drosophila* brain sections of *elav*-Gal4-derived flies were analysed using the amyloid-specific dye p-FTAA (Aslund et al., 2009) (Fig. 5). In concordance with previous studies (Jonson et al., 2015), extensive formation of amyloid aggregates were found in the flies expressing the Aβ_1-42_ peptide. A smaller, but detectable, amount of p-FTAA-positive aggregates were found in the AβPP-BACE1 flies. No p-FTAA-positive signal was detected in control, AβPP or BACE1 flies. In the Aβ_1-42_×2 flies, the amyloid aggregates were found spread out in almost the entire fly brain while the amyloid aggregates detected in the AβPP-BACE1 flies were limited to a smaller area.

## DISCUSSION

Neurodegenerative diseases belong to one of the most devastating disease groups and involve the progressive loss of a specific population of neurons characteristic for each disease type. Most of these conditions are fatal due to the lack of mechanism-based therapeutic strategies to halt the degeneration process. In the search to increase our understanding of disease mechanisms and to find effective treatment strategies, *Drosophila* have been shown to provide an important resource to study neurodegenerative diseases, including AD, Parkinson's and Huntington's disease (Crowther et al., 2006; Marsh et al., 2003; Whitworth, 2011). The power of the fly model lies in the highly sophisticated toolbox that is available to manipulate the fly genome, allowing the design of transgenic flies where the transgene can be ectopically expressed in a specific tissue. The fly model is also convenient to use in large-scale screens for therapeutic compounds and to perform genetic screens to dissect cellular pathways involved in the disease.

The genetic base for AD was elucidated after links between familial AD patients and mutations in the *AβPP* gene were found (Kang et al., 1987; Selkoe et al., 1987). Several of these mutations occur in the Aβ peptide, the cleavage product produced by the processing of AβPP with β-secretase followed by γ-secretase, and a clear positive correlation has been found between the ability of the peptide to form prefibrillar aggregates and cytotoxicity (Brorsson et al., 2010; Göransson et al., 2012; Luheshi et al., 2007). The longer and more hydrophobic Aβ_1-42_ peptide is more likely to form prefibrillar aggregates compared to the shorter and less hydrophobic Aβ_1-40_ peptide (Dahlgren et al., 2002), and in the sporadic form of AD, it is known that an increased ratio of Aβ_1-42_ to Aβ_1-40_ correlates with an increased risk of subsequently developing AD (Kumar-Singh et al., 2006). Thus, a lot of attention has been focused on exploring the toxic effects of the Aβ_1-42_ peptide to map the mechanism of Aβ proteotoxicity. During the last decade, several studies have employed *Drosophila* as a model to study AD using one of two approaches; either the Aβ peptides are fused to a secretion sequence and directly produced from transgenes or the Aβ peptides are produced by the processing of human AβPP (Caesar et al., 2012; Chakraborty et al., 2011; Mhatre et al., 2014). Commonly used phenotypic indicators for neurodegeneration in AD flies are reduced longevity and deficient locomotor activity, which result when genes related to the disease are over-expressed in the CNS of flies using the *elav*-Gal4 driver (Dias-santagata et al., 2007; Feany and Bender, 2000). Another phenotypic indicator for cell toxicity is the so-called rough eye phenotype, which occurs when disease-related genes are overexpressed in the fly retina during eye development using the *gmr*-Gal4 driver (Crowther et al., 2005; Kumita et al., 2012; Tare et al., 2011). This phenotype corresponds to the loss of retinal cells, including photoreceptors, and result in disruption of the eye structure that can be detected at the day of eclosion. *Drosophila* studies based on the direct expression of Aβ peptides from transgenes or on the production of peptides by AβPP processing have shown neurotoxic effects such as reduced longevity and locomotor dysfunction, the rough eye phenotype, learning deficits and the accumulation of extracellular deposits, revealing that AD-like neurodegeneration can be linked to the production of the Aβ_1-42_ peptide. These results suggest these fly models to be useful to study AD pathogenesis (Chakraborty et al., 2011; Crowther et al., 2005; Greeve et al., 2004; Iijima et al., 2004).

In this study, two different *Drosophila* AD models (Aβ_1-42_-expressing flies and AβPP-BACE1 co-expressing flies) were analysed in parallel and compared with respect to toxic effects (assessed by the eye phenotype and by longevity and locomotor assays) and to levels of the Aβ_1-42_ peptide to evaluate how the toxic effects might be linked to the level of Aβ_1-42_ and/or how the peptide is generated. In the rough eye phenotype analysis, a clear disruption in the arrangement of ommatidia was detected in the AβPP-BACE1 flies, revealing a toxic effect during eye development. MSD analyses showed the presence of both sAβPP_β_ and the Aβ_1-42_ peptide in these flies, confirming correct processing of AβPP by BACE1 and fly γ-secretase. For the Aβ_1-42_ expressing flies, a disruption in the eye structure could be detected for isolated ommatidia in line with previous analyses (Crowther et al., 2005), however, the statistical analysis did not show any significant difference in the eye structure between the Aβ_1-42_ flies and control flies. MSD analysis confirmed the production of the Aβ_1-42_ peptide in these flies, and the level was found to be approximately 1000 times higher than the Aβ_1-42_ level detected in the AβPP-BACE1 flies. Thus, these data show that the toxic effect on eye development does not correspond to the amount of detected Aβ_1-42_ in the flies because the eye phenotype of the AβPP-BACE1 flies was more severe compared to that of the Aβ_1-42_ flies despite the lower level of Aβ_1-42_ that was found in the AβPP-BACE1 flies.

When analysing the toxic effects via longevity and locomotor assays, both fly models showed neurodegenerative behaviour; the Aβ_1-42_ and the AβPP-BACE1 flies had significantly reduced median survival time and showed reduced velocity and a higher degree of disorientation than the corresponding control flies. Also, in the longevity assay a small reduction in the median survival time was detected for both the AβPP and BACE1 flies compared to control flies (2 and 5 days, respectively). However, since the reduction in the median survival time for the AβPP-BACE1 flies compared to control flies was found to be 16 days, it is clear that the detrimental effects on the median survival observed for the AβPP-BACE1 flies result from co-expression of AβPP and BACE1 and not due to the slight reduction in the median survival time observed when individually expressing AβPP or BACE1 in the fly CNS.

The longevity assay showed that the AβPP-BACE1 flies had a significantly reduced median survival compared to the Aβ_1-42_×2 flies and the MSD analyses of the Aβ_1-42_ level revealed the same finding discovered in the eye phenotype experiment; that the Aβ_1-42_ level was significantly higher in the Aβ_1-42_×2 flies compared to the AβPP-BACE1 flies, both in the head (40 times) and in the body (56 times). These data clearly showed that the toxic effect per amount of detected Aβ_1-42_ peptide was higher for the AβPP-BACE1 flies compared to the Aβ_1-42_ flies. The data also revealed that in both AD fly models, a large portion of the detected Aβ_1-42_ was transported from the head to the body. This was also found for the sAβPP_β_ detected in the AβPP-BACE1 flies; a higher level of the protein was found in the body compared to the head.

We also investigated formation of AD characteristic amyloid aggregates in the AβPP-BACE1 flies as well as in the Aβ_1-42_×2 flies using the amyloid aggregate specific dye p-FTAA (Aslund et al., 2009). Large p-FTAA-positive aggregates were detected in the Aβ_1-42_×2 flies while smaller p-FTAA-positive aggregates were found in the AβPP-BACE1 flies. Aggregates in both AD fly lines were found surrounding cell nuclei in the fly brain. In the Aβ_1-42_×2 flies, the amyloid aggregates were found spread out in the entire fly brain, while the amyloid aggregates in the AβPP-BACE1 flies were limited to a smaller area. Clearly, the Aβ aggregate load does not correlate to the *in vivo* Aβ_1-42_ proteotoxic effect since more Aβ aggregates were found in the Aβ_1-42_ flies compared to the AβPP-BACE1 flies while the Aβ_1-42_ proteotoxic effect was higher for the AβPP-BACE1 flies. In humans, amyloid plaques have been found in brains of non-AD patients (Lue et al., 1996), indicating that to develop the AD characteristic symptoms due to potential Aβ proteotoxicity, more than just the formation of amyloid aggregates is required.

We can conclude that despite the fact that the Aβ_1-42_ peptide can be generated in two different ways (direct production and secretion of the peptide or production of the peptide by AβPP processing), both *Drosophila* AD models exhibited neurodegeneration that could be linked to the production of the Aβ_1-42_ peptide in the fly. However, levels of the peptide were found to be considerably lower in the AβPP-BACE1 flies compared to the Aβ_1-42_ flies. Apparently, when the peptide is directly produced and secreted, a much higher level of Aβ_1-42_ is required to achieve a similar degree of toxicity as that achieved when the Aβ_1-42_ peptide is produced by AβPP processing. Thus, it is clear that Aβ_1-42_ proteotoxicity is more dependent on how and where the peptide is being generated in the flies rather than on the total amount produced. This is very important knowledge because if we want to find a way to fight Aβ proteotoxicity, we need to know how the peptide exerts its proteotoxic effects. Does it happen inside the cells in certain compartments where critical concentrations of the Aβ peptide can accumulate to form toxic aggregates, or does it happen outside the cells as a result of prefibrillar species formed *de novo* or by secondary nucleation (Cohen et al., 2013)?

The processing of AβPP occurs at the cell membrane, but the activity of BACE1 has been found to be particularly high in endosomes that are about to fuse with lysosomes for final degradation of AβPP (Haass et al., 1992; Huse et al., 2000). The decreased pH and limited space of these vesicles provide ideal conditions for the Aβ peptide to aggregate if accumulation of the peptide occurs faster than its degradation. In this situation, even a small amount (picomolar concentrations) of accumulated Aβ peptides might be enough to cause fatal damage to the cell (Zheng et al., 2009; Zheng et al., 2011). On the other hand, it is known that aggregated forms of the Aβ_1-42_ peptide possess cytotoxic properties when exposed to cells at nano- to micromolar concentrations *in vitro*, revealing a direct toxic action of the Aβ_1-42_ peptide on the cell surface (Göransson et al., 2012).

In addition to the production of sAβPPβ, Aβ1-42 and CTFs in the AβPP-BACE1 flies, other Aβ isoforms with different lengths and post-translationally modified N-terminal truncated variants of the Aβ peptide are most likely also produced when AβPP is processed in the flies, as has been found in studies in AβPP-overexpressed cell lines and in brain tissue from AD patients (Portelius et al., 2010, 2013). The *in vivo* proteotoxcicity of different N- and C-terminally truncated variants of the Aβ peptide was investigated using *Drosophila* (Jonson et al., 2015)*.* The N-truncated Aβ variants (Aβ_3-42_ and Aβ_11-42_) had a pronounced toxic effect comparable with the effect observed for Aβ_1-42_ while the C-terminally truncated versions of Aβ (1-40, 1-39, 1-38, 1-37) were non-toxic. It has also been shown that Aβ extracted from cell media and from AD brains has greater cytotoxic effects compared to synthetic Aβ_1-42_ peptide, indicating that the combination of the different Aβ isoforms that are formed during AβPP processing is more toxic than a single Aβ isoform. Thus, the toxic effects observed in the AβPP-BACE1 flies cannot be attributed solely to the Aβ_1-42_ peptide but may also be due to the combined toxic effects caused by the various Aβ isoforms that can be produced when AβPP is processed *in vivo*. Indeed, a considerable amount of the Aβ_1-40_ peptide was detected in the AβPP-BACE1 flies. Furthermore, the level of the Aβ_1-40_ peptide was higher compared to the level of the Aβ_1-42_ peptide in both the head (Aβ_1-42_:Aβ_1-40_ ratio 3:4) and in the body (Aβ_1-42_:Aβ_1-40_ ratio 1:2). In a study by Pauwels et al. (2012) it was demonstrated that different ratios between Aβ_1-42_ and Aβ_1-40_ resulted in alterations in the formation process of oligomeric species effecting the Aβ neurotoxicity. In another study (Kuperstein et al., 2010) an Aβ_1-42_:Aβ_1-40_ ratio of 3:7 resulted in increased nucleation and aggregation kinetics, while the fibril formation was delayed, indicating that at this Aβ_1-42_:Aβ_1-40_ ratio the formed prefibrillary species are retained in their oligomeric state compared to those prefibrillary species that are solely formed from the Aβ_1-42_ peptide. Thus, the co-existence of Aβ_1-42_ and Aβ_1-40_ in the AβPP-BACE1 flies could be of significant importance to the neurotoxic effect detected in these flies.

The physiological effect of other cleavage products of AβPP, such as both sAβPP_β_ and sAβPP_α_, has been found to be involved in neurite outgrowth and neuronal proliferation (Chasseigneaux et al., 2011) as well as in triggering apoptosis in peripheral neurons (Nikolaev et al., 2009). In this study, high levels of sAβPP_β_ were detected in the body of the AβPP-BACE1 flies when the *elav*-Gal4 construct was used to direct protein expression to the CNS of the fly. The level of sAβPP_β_ in the head was considerably lower and did not differ significantly from the level of sAβPP_β_ detected in the AβPP fly heads. Thus, the toxic effects observed in the longevity and locomotor assays cannot be attributed to detrimental effects caused by the sAβPP_β_ fragment since our data reveal that the sAβPP_β_ generated in the AβPP-BACE1 flies is efficiently transported from the head to the body of the flies. However, when using the *gmr*-Gal4 construct, which directs the protein expression to the retina of the flies, a significant higher level of sAβPP_β_ was detected in the heads of the AβPP-BACE1 flies compared to the AβPP flies. It is therefore possible that a potential toxic effect from the sAβPP_β_ fragment contributes to the rough eye phenotype observed for the AβPP-BACE1 flies.

In the AβPP-BACE1 flies, production of the Aβ peptide can occur by the processing of AβPP at the outer cell membrane, which results in the release of sAβPP_β_ and Aβ peptides to the extracellular space, and/or inside the cells at the membrane of late endosomes, which results in the release of sAβPP_β_ and Aβ peptides into endosomal compartments. Our hypothesis is that the toxic effects in the AβPP-BACE1 flies are caused by the Aβ peptides that are produced in the late endosomes, allowing proteotoxic effects to occur at the low Aβ_1-42_ peptide levels detected in the heads of the *gmr*-Gal4- and *elav*-Gal4-derived flies (7 pg/fly and 0.8 pg/fly, respectively). We further speculate that the fraction of the Aβ_1-42_ peptide detected in the body of the *elav*-Gal4-derived AβPP-BACE1 flies (0.95 pg/fly) corresponds to Aβ peptides that are generated on the cell surface and released to the extracellular space and/or generated in endosomal compartments and secreted and eventually transported from the head to the fly body without causing any harm to the fly neurons. In the Aβ_1-42_ flies, the Aβ peptide is directly produced and secreted via the endoplasmatic reticulum (ER) and Golgi apparatus. We believe that the toxic effects in this fly model are caused by the high amount of Aβ peptides located in the head of the *gmr*-Gal4- and *elav*-Gal4-derived flies (7900 pg/fly and 32 pg/fly, respectively). If properly secreted, these Aβ levels might be high enough to cause the formation of extracellular aggregates, which are toxic to the cells. This hypothesis is in line with the study of Hermansson et al. (2014), where the rescue mechanism of BRICHOS in Aβ_1-42_ flies was suggested to be due to the binding of BRICHOS to extracellularly located Aβ_1-42_ aggregates and fibrils. This binding blocked secondary nucleation, whereby Aβ monomers associate with the surface of already existing fibrils and form new oligomers, some of which are toxic (Cohen et al., 2013). However, in the study by Crowther et al. (2005), intracellular aggregates of the Aβ_1-42_ peptide were detected and therefore, the potential toxic effects of Aβ_1-42_ aggregates that have formed inside the cells (in ER or Golgi) or that have been taken up by the cells cannot be ruled out in this fly model. Nevertheless, our data clearly show that a large portion of the Aβ peptide is secreted and then transported from the heads to the bodies of the flies; this transport likely occurs without causing any damage to the fly neurons.

In conclusion, the toxic effects revealed in these two fly models seem to have different mechanisms. A low level of Aβ_1-42_ is enough to produce toxic effects in the AβPP-BACE1 flies; these toxic effects most likely result from Aβ peptides that are generated within cellular compartments. In contrast, a much higher level of Aβ_1-42_ is needed to achieve toxicity in the Aβ_1-42_ flies. Thus, it is clear that Aβ proteotoxicity is highly dependent on how and where the peptide is produced in the flies rather than on the overall Aβ levels detected. Furthermore, it is necessary to take into consideration that the AβPP-BACE1 flies do not produce only a single isoform of the Aβ peptide; instead, a wide range of different cleavage products are formed from AβPP processing as well as post-translationally modified Aβ isoforms. Indeed, the co-existence of Aβ_1-42_ and Aβ_1-40_ detected in the AβPP-BACE1 flies could play a significant role to the neurotoxic effect detected in these flies. Therefore, it is important to keep in mind the difference between studying the effect of a single Aβ isoform in the Aβ fly model and studying the effect of AβPP processing *in vivo* in the AβPP-BACE1 fly model when using *Drosophila* models to investigate disease mechanisms or therapeutic strategies in AD research.

## MATERIALS AND METHODS

### Drosophila stocks

The Gal4/UAS system was used for tissue specific expression in UAS transgenic *Drosophila melanogaster* (Brand and Perrimon, 1993). Two different tissue specific driver strains were utilised; the *elav*-Gal4 driver, which directs protein expression to the CNS of the fly, and the *gmr*-Gal4 driver, which directs protein expression to the photoreceptors of the fly retina. Control *w^1118^* flies and different fly strains expressing human AβPP or human BACE1 were purchased from the Bloomington Stock Center. The AβPP and BACE1 strains that showed the highest expression levels, as analysed by western blot, were chosen to design a novel fly strain in which AβPP and BACE1 were co-expressed (the AβPP-BACE1 fly). Aβ_1-42_ flies were kindly provided by D. Crowther (AstraZeneca, Floceleris, Oxbridge Solutions Ltd.). The fly crosses were set up at 18°C (*elav*-Gal4) or at 25°C (*gmr*-Gal4) at 60% humidity with 12:12 h light:dark cycles. For protein quantification and immunohistochemistry, flies were aged for 7 days at 29°C (*elav*-Gal4) or snap-frozen on the day of eclosion (*gmr*-Gal4). The rough eye phenotype analysis was performed using the *gmr*-Gal4 driver, and the flies were analysed at the day of eclosion. The longevity and locomotor assays were performed using the *elav*-Gal4 driver, and the flies were maintained at 29°C after eclosion.

### Scanning electron microscopy

At the day of eclosion, the flies (from crosses using the *gmr*-Gal4 driver) were collected and euthanized using ether. The flies were left to air dry for 24 h before coating with platinum. The eye phenotypes were analysed using a LEO 1550 Gemini scanning electron microscope. The images were taken at 350× magnification. In a blinded set-up, the images of the eyes of each genotype were printed and assigned a square containing approximately 100 ommatidia in the centre of the eye. All ommatidia within this square were calculated and the number of abnormal ommatidia was related to the total number of ommatidia in the square. *n*≥4 flies per genotype.

### Western blot

Flies expressing AβPP and BACE1 individually or concurrently under the influence of the *elav*-Gal4 driver were snap-frozen after being aged for 7 days at 25°C. Forty heads from each genotype were homogenized in 50 μl RIPA buffer containing a protease inhibitor (Complete EDTA-free Protease Inhibitor Cocktail Tablets, Roche Diagnostic). The homogenate was centrifuged for 10 min at 15,000 ***g***, and the supernatant was collected. To account for differences in protein content due to the extraction step, a protein quantification assay was performed on all fly homogenates using the Bio-Rad DC Protein Assay Kit II (5000112, Bio-Rad). NuPAGE LDS sample buffer (4×) (NP008, Life Technologies) and NuPAGE sample reducing agent (10×) (NP009, Life Technologies) were added to each sample, which were then heated at 70°C for 10 min. Gel electrophoresis was performed using Bolt 4-12% Bis-Tris Plus gels (NW04120BOX, Life Technologies). Transfer was performed using an original iBlot^®^ Gel Transfer Device from Life Technologies. The primary antibodies included 6E10, an Aβ monoclonal antibody (1:10,000; Sig-39340-200, Covance, Inc.); an AβPP C-terminal antibody (1:7000; A8717, Sigma Aldrich); and a BACE1 antibody (1:2000; ab2077, Abcam). Tubulin was used as a loading control (1:3000; ab7291, Abcam). *n*=4 (with 40 flies in each set).

### Sample preparation for protein quantification assays

The flies were snap-frozen using liquid nitrogen on the day of eclosion (*gmr*-Gal4) or after ageing for 7 days at 29°C (*elav*-Gal4). Approximately 20 fly heads or bodies were homogenized in 25 or 60 μl extraction buffer [50 mM HEPES, 5 M guanidinium chloride, 5 mM EDTA, 1× protease inhibitor (Complete EDTA-free Protease Inhibitor Cocktail Tablets, Roche Diagnostics)], respectively for extraction of both soluble and insoluble proteins. After homogenization, the samples were incubated for 10 min at room temperature (RT), followed by 4 min of sonication. The homogenates were centrifuged (15,000 ***g***, 5 min), and the supernatant was collected. The supernatant was diluted 10× in Diluent 35 (for quantification of Aβ_1-42_) or 1% Blocker A (diluted in Tris wash buffer) (for quantification of sAβPP_β_) and stored at −80°C. To account for differences in the protein extraction step, the total amount of protein extracted was quantified using the Bio-Rad DC Protein Assay Kit II (500-0112, Bio-Rad).

### Quantification of Aβ species by MSD analysis

For the analysis of total Aβ_1-42_ (*gmr*-Gal4) a multi-spot 96-well V-PLEX human Aβ_1-42_ kit plate (K151LBE-1, Meso Scale Discovery) was used. For analysis of total Aβ_1-42_, Aβ_1-40_ and Aβ_1-38_ a V-PLEX Aβ Peptide Panel 1 (6E10) Kit plate (K15200E-1, Meso Scale Discovery) was used. The wells in each plate were blocked for 1 h by adding 150 μl Diluent 35 at RT with gentle agitation. After blocking, 50 μl of each prepared protein sample (*gmr*-Gal4) was added to the plate in triplicate (1 h, RT, gentle agitation). The wells were then washed three times using 150 μl PBS-T before adding 25 μl of the detection antibody (50× Sulfo tag 6E10, Meso Scale Discovery) (1 h, RT, gentle agitation). For the analysis of multiple Aβ species, 25 μl protein sample and 25 μl of detection antibody was added to the wells (2 h, RT, gentle agitation). The wells were then washed using PBS-T (3×150 μl), and 150 μl 2× reading buffer was added (10 min of incubation at RT, no agitation). The plate was analysed using a SECTOR Imager 2400 instrument (Meso Scale Discovery). *n*=3 for *elav*-Gal4 and *n*=3 for *gmr*-Gal4.

### Quantification of soluble AβPP_β_ by MSD analysis

The wells in a multi-spot 96-well sAβPP_β_ kit plate (K151BTE-1, Meso Scale Discovery) were blocked for 1 h by adding 150 μl 3% Blocker A (diluted in Tris wash buffer) at RT with gentle agitation. After blocking, 25 μl of each prepared protein sample was added to the plate in triplicate (1 h, RT, gentle agitation). The wells were washed using 3×200 μl Tris wash buffer before 25 μl detection antibody was added to each well (50× Sulfo tag anti-sAβPP_β_, Meso Scale Discovery) (1 h, RT, gentle agitation). The wells were then washed using Tris wash buffer (3×200 μl), followed by the addition of 150 μl reading buffer (10 min of incubation at RT, no agitation). The plate was analysed using a SECTOR Imager 2400 instrument (Meso Scale Discovery). *n*=5 for *elav*-Gal4 and *n*=3 for *gmr*-Gal4.

### Longevity assay

Fly crosses using the *elav*-Gal4 driver were set up at 18°C and maintained at 29°C (60% humidity, 12:12 h light:dark cycle) after eclosion. A set of 100 female offspring for each genotype was collected on the day of eclosion. The flies were divided into groups of approximately 20 flies and placed in plastic vials containing agar food (20 g sugar, 20 g agar-agar/l H_2_O) and yeast paste (dry baker's yeast mixed with water). Every 2-3 days, the flies were transferred into new vials containing fresh agar food and yeast paste, and the number of living flies was counted. This was repeated until all the flies had died. GraphPad Prism software 6 (GraphPad Software) was used to generate Kaplan–Meier survival curves (Kaplan and Meier, 1958).

### Locomotor assay

Fly crosses using the *elav*-Gal4 driver were set up at 18°C and maintained at 29°C (60% humidity, 12:12 h light:dark cycle) after eclosion. Sets of 30 female offspring for each genotype were collected on the day of eclosion, divided into groups of 10 and placed in narrow vials containing agar food and yeast paste. To analyse the flies' locomotor behaviour, the flies were tapped to the bottom of the vial and filmed for 90 s. Every 30 s, the flies were tapped to the bottom of the vial (re-activating locomotor behaviour), generating three video clips with a total of nine 30-s clips for each genotype. This was carried out every 2-3 days and included transfer of the flies into new vials containing fresh agar food and yeast paste. The videos were processed and analysed using iFly software (Jahn et al., 2011), which calculated the velocities and angles of movement of the flies recorded in each clip. The data were plotted using GraphPad Prism 6.

### p-FTAA staining

*Drosophila* heads (*elav*-Gal4) were embedded in Tissue-Tek OCT compound (Histolab) using Cryomold-specimen moulds and stored at −80°C until use after being aged for 7 days in 29°C. The OCT blocks were sectioned using a Microm HM 550 Cryostat (Microm International GmbH) into 10 μm thin sections that were placed on Superfrost Plus slides (Menzel-Gläser) and stored at −20°C until use. The sections were fixed for 10 min at room temperature using 90% ethanol, and then rehydrated in 75, 50 and 0% ethanol in 2 min steps followed by a washing step (3×3 min, PBS). Following the washing, the sections were incubated with 3 μM of the amyloid-specific dye p-FTAA (Aslund et al., 2009) (30 min, RT). After incubation with p-FTAA, the washing step was repeated. For visualization of cell nuclei, the sections were incubated with 5 μM ToPro3 (TO-PRO-3, Life Technologies) (15 min, RT). The washing step was repeated, followed by a final wash using dH_2_O. The sections were left to dry in RT, followed by mounting using DAKO mounting medium (DAKO #S3023; DAKO), and stored at 4°C. The brain sections were analysed using a LSM 780 confocal microscope (Zeiss). All micrographs were processed in the same way using Photoshop (Adobe). A minimum of eight fly brains were analysed for each genotype, representative images are shown.

### Statistical analysis

The data were analysed using IBM SPSS Statistics for Macintosh, Version 23.0 or GraphPad Software. One-way ANOVA followed by Tukey's test or the Kruskal–Wallis test was used to identify differences in the number of abnormal ommatidias and protein levels. GraphPad Prism software 6 was used to generate Kaplan–Meier survival curves (Kaplan and Meier, 1958).
